# Right Ventricular Metastasis of Ewing Sarcoma Treated Through Surgical Resection

**DOI:** 10.1016/j.jaccas.2024.102654

**Published:** 2024-12-04

**Authors:** Toshiki Yokoyama, Hiroshi Kurazumi, Ryosuke Nawata, Kazumasa Matsunaga, Ryo Suzuki, Akihito Mikamo, Kimikazu Hamano

**Affiliations:** Division of Cardiac Surgery, Department of Surgery and Clinical Science, Yamaguchi University Graduate School of Medicine, Ube, Yamaguchi, Japan

**Keywords:** cancer, chordae, papillary muscles, tricuspid valve, valve repair

## Abstract

A 27-year-old man diagnosed with right ventricular metastasis of Ewing sarcoma was referred to our institution. We surgically resected the metastatic tumor to prevent sudden death and reconstructed the right ventricle and tricuspid valve. He has survived for 1 year postoperatively without recurrence.

## Graphical Abstract

**Figure:**
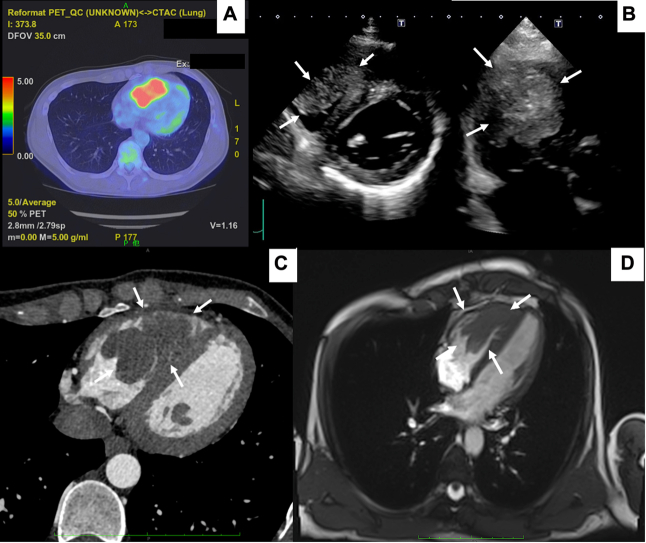

## History of Presentation

A 27-year-old male patient had a history of Ewing sarcoma and a primary right femoral lesion. He presented to us for treatment of a tumor in his right ventricle. He was walking with a cane in the aftereffects of leg surgery and was considered to have a Clinical Frailty Scale score of 4.

**Take-Home Messages**

• This case highlights the importance of multimodality imaging in assessing metastatic cardiac tumors, particularly in determining the extent of the disease and guiding the surgical approach for complex resections.
• Even a huge metastatic lesion occupying the right ventricle can be surgically removed, with tricuspid valve reconstruction, potentially preventing sudden death.

## Past Medical History

Seven years prior, he presented with a tumor in his right hip joint, which was diagnosed as Ewing sarcoma based on biopsy examination. After neoadjuvant chemotherapy, he underwent extensive tumor resection, external radiation therapy, and total hip arthroplasty. Moreover, he underwent postoperative chemotherapy. One year prior, positron emission tomography-computed tomography (PET-CT) had revealed fluorodeoxyglucose (FDG) accumulation in the upper left lung lobe. He underwent a thoracoscopic partial pneumonectomy of the left lung lesion, with subsequent pathologic examination revealing that the nodule was a metastasis of Ewing sarcoma.

## Differential Diagnosis

There was a possibility that the tumor could have been a primary tumor in the heart or a metastasis from another part of the body. Based on the patient’s medical history, however, the tumor was diagnosed as metastasis of Ewing sarcoma.

## Investigation

**Figure 1:**
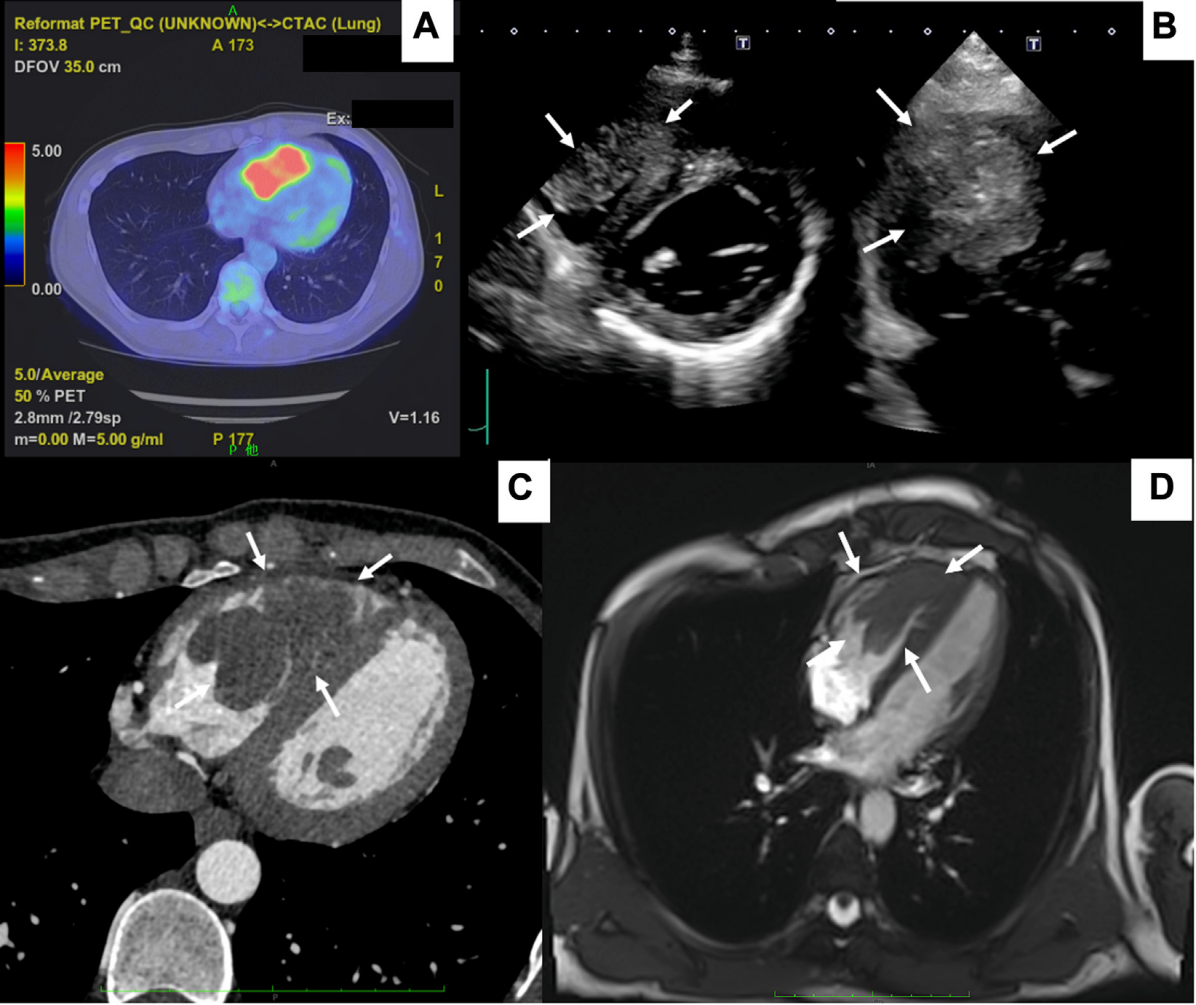
Multi-Modality Imaging of the Right Ventricle (A) Positron emission tomography–computed tomography showing fluorodeoxyglucose accumulation in the right ventricle. (B) Preoperative echocardiography. (C) Contrast-enhanced computed tomography revealing a low-density mass in the right ventricle. (D) Short T1 inversion recovery mode of cardiac magnetic resonance displaying a low-density mass in the right ventricle. White arrows indicate the metastatic tumor.

PET-CT performed 6 months after lung surgery revealed abnormal FDG accumulation (maximum standardized uptake value: 9.3) in the right ventricle (Figure 1A). Echocardiography revealed a mobile, heterogeneous mass (21 × 32 × 62 mm) with an irregular surface in the right ventricular cavity (Figure 1B, Video 1). Contrast-enhanced CT showed a low-absorption right ventricular mass (Figure 1C). Cardiac magnetic resonance under short T1 inversion recovery conditions revealed a low-intensity lesion extending from the ventricular septum to the apical myocardium (Figure 1D).

## Management

Surgical resection was performed to prevent tumor embolism or sudden death caused by tumor incarceration. At the time of surgery, no evidence of metastasis was found beyond the heart. Specifically, cardiac access was achieved through a median sternotomy. Subsequently, cardiopulmonary bypass was performed via ascending aortic and bicaval cannulation; moreover, cardiac arrest was achieved using antegrade cold blood cardioplegia. The free right ventricular wall was incised and extended to the outflow tract; subsequently, we could directly visualize an irregular surface of the tumor.

**Figure 2:**
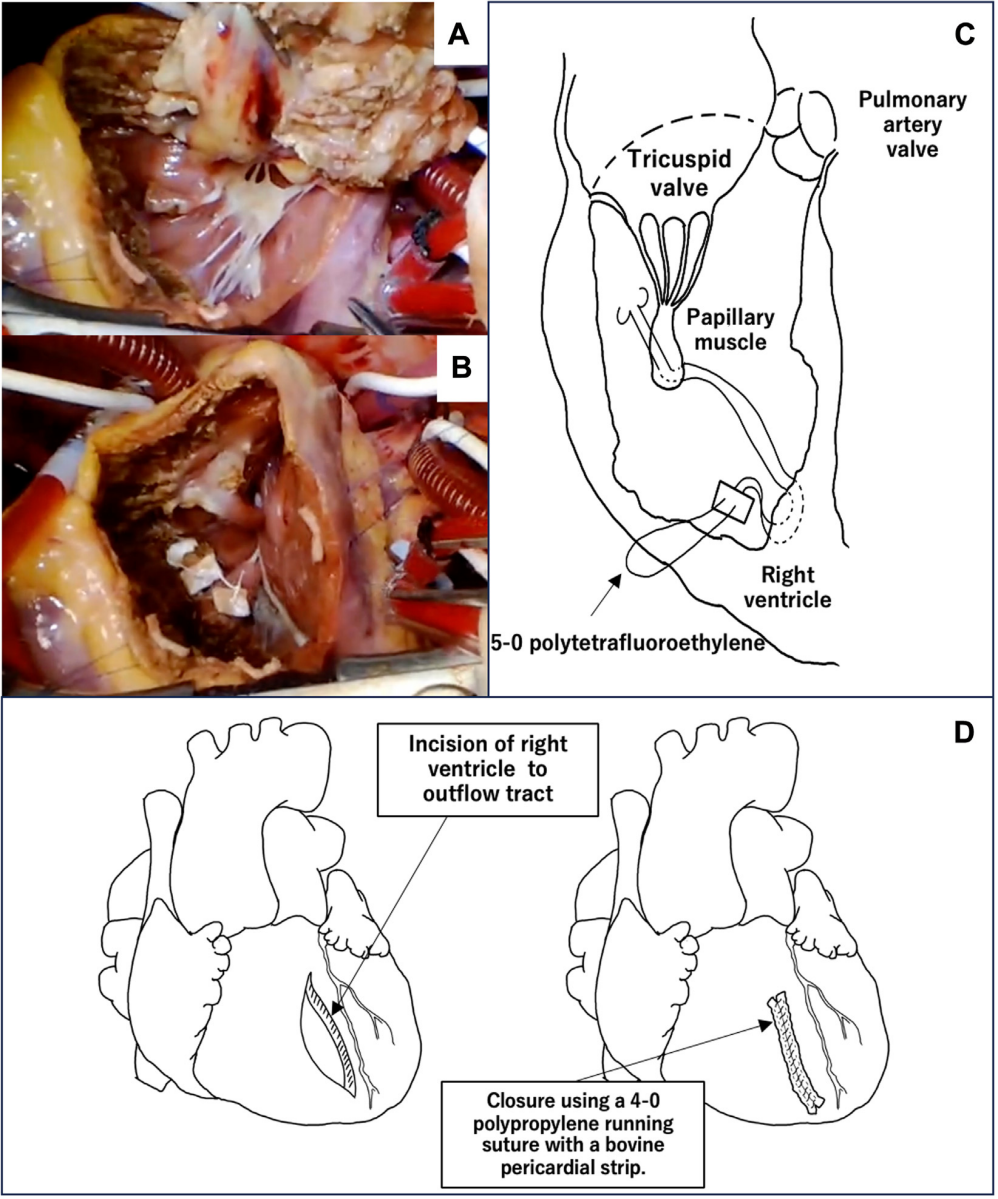
Operative Findings (A) The right ventricular wall and part of the ventricular septum were resected with the tumor. (B, C) The papillary muscles of the tricuspid valve during reconstruction. The papillary muscle of the tricuspid valve was stitched to the right ventricular myocardium. (D) The incision of the right ventricle was closed with a bovine pericardial strip.

A clear border between the tumor and myocardium was observed. There was broad tumor invasion into the right ventricular septum, anterior wall of the right ventricle, and papillary muscle of the tricuspid valve (Figure 2A). We performed resection of the entire tumor with right ventricle structures. The remnant papillary muscles were reconstructed with an artificial chord using 5-0 polytetrafluoroethylene (Figures 2B and 2B). The right ventricular incision was closed using a 4-0 polypropylene running suture with a bovine pericardial strip (Figure 2D). Additionally, the right atrium was incised to evaluate the residual tricuspid valve regurgitation. Tricuspid valve prolapse and regurgitation were observed. We placed stitches at the tricuspid valve using a 5-0 polypropylene horizontal mattress suture; moreover, control of regurgitation was achieved. The patient was easily weaned from cardiopulmonary bypass. The operative findings are shown in Video 1.

Pathologically, the tumor’s histology was similar to that of the primary tumor and pulmonary metastases in the femur. The cardiac tumor was diagnosed as a right ventricular metastasis of Ewing sarcoma.

## Outcome and Follow-Up

**Figure 3:**
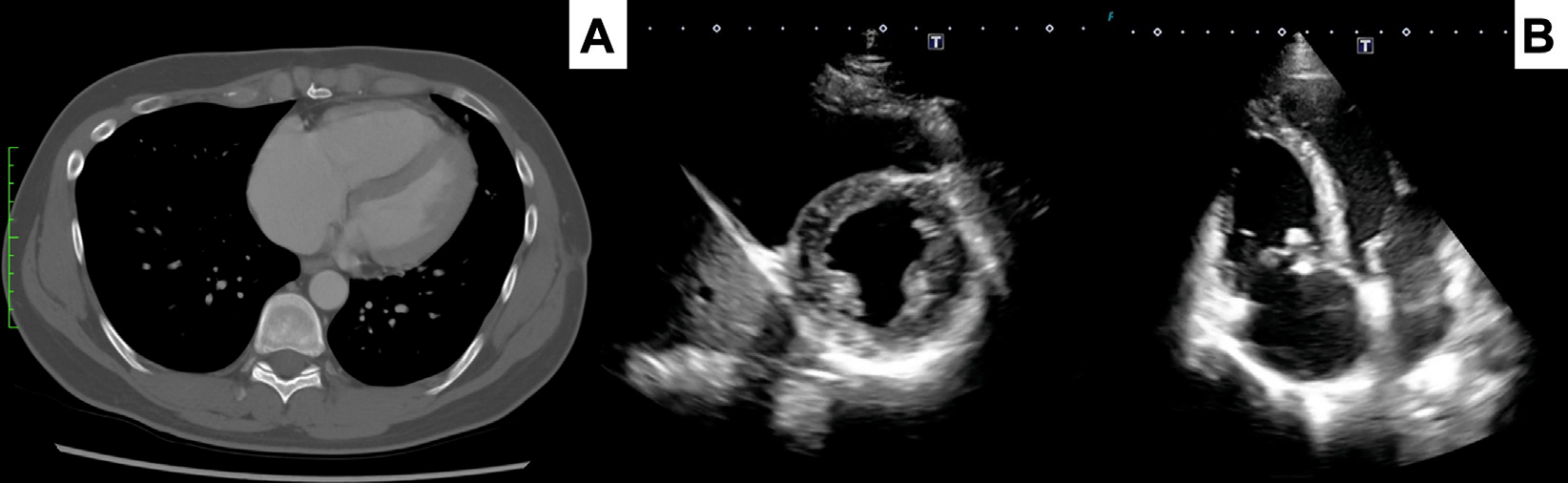
Postoperative Image of the Right Ventricle (A) Postoperative computed tomography findings. (B) Postoperative transthoracic echocardiography showing no residual tumor in the right ventricle.

The patient was discharged on postoperative day 14. His postoperative Clinical Frailty Scale score was 4. Postoperative echocardiography showed moderate tricuspid regurgitation, whereas both echocardiography and CT confirmed the absence of any residual tumor (Figure 3, Video 1). After discharge, he underwent additional chemotherapy. One year after surgery, tumor recurrence has not occurred. Tricuspid regurgitation remained moderate, and cardiac decompensation was not detected.

## Discussion

Ewing sarcoma is the second most common bone and soft tissue malignancy in children and young adults; moreover, 85% of patients have a t(11;22) (q24:q12) chromosomal translocation. The resulting EWS-FLI-1 fusion protein may contribute to the development of Ewing sarcoma.^1^ There have been advances in the treatment of Ewing sarcoma, with survival rates improving from 10% before chemotherapy introduction to 75% in current patients with localized tumors.^2^ However, the survival rate remains low in the presence of metastases, with a reported 5-year survival rate of 25%.^3^

There have been a few reported cases of Ewing sarcoma of cardiac origin; however, it is extremely rare, and its incidence is unknown. Surgical resection has remained the standard curative treatment for primary malignant cardiac tumors. Regarding the treatment of metastatic cardiac tumors, if the metastasis is limited to the heart and the primary tumor lesion is well controlled, the primary goal is complete resection as a curative surgery.^4^ The primary lesion in our case was well controlled, and the metastasis was limited to the heart alone. Owing to the large size of the right ventricular tumor, surgical resection was speculated to be helpful in preventing tumor embolism or sudden death caused by tumor incarceration. There have been a few cases of surgical resection of right ventricular metastases of Ewing sarcoma. Datrice et al^1^ encountered a metastatic lesion sized 1.4 × 4 cm; because this tumor was not too large, it was simply resected. Marija et al^5^ also performed a simple surgical resection of a metastatic lesion sized 2.6 × 3.5 cm without mentioning any concurrent tricuspid valve repair. In our case, tricuspid valve reconstruction was required because the tumor was large and had broadly invaded the right ventricle. However, the postoperative course remained uneventful.

An alternative therapeutic option is neoadjuvant chemotherapy; it may allow surgeons to minimize the extent of surgical resection. Subroto et al^6^ reported a case of a cardiac primary Ewing sarcoma invading the mediastinum; the tumor, which seemed unresectable before neoadjuvant chemotherapy, could be successfully resected after the patient received chemotherapy. In our case, the tumor expanded to fill the entire right ventricle within 6 months after lung surgery. Excessive tumor growth poses a significant risk of sudden death, classifying the condition as an oncologic emergency. Accordingly, we did not have sufficient time for administering additional chemotherapy.

For systemic screening in cases of Ewing sarcoma, PET-CT, magnetic resonance imaging, and CT are commonly used, with FDG-PET being particularly effective for detecting distant metastases. However, CT has demonstrated superiority over PET-CT in identifying lung lesions.^2^ In our case, no cardiac tumor was detected on CT or PET-CT at least 6 months prior, but both modalities successfully identified it at the time of diagnosis. This leads to the hypothesis that CT and PET-CT may have comparable accuracy in detecting cardiac metastases. Furthermore, echocardiographic findings in our case indicated tumor invasion of the ventricular septum and right ventricular wall. Therefore, multimodality imaging is essential to accurately assess the extent of the tumor, guide the surgical approach, and determine the feasibility of reconstruction.

When resecting large cardiac tumors, it is difficult to maintain sufficient resection margins. Furthermore, the cardiac function must be maintained postoperatively. In our case, a tricuspid valve repair was required to maintain the postoperative cardiac function. The pathologic findings revealed residual tumor cells very close to the margin on the ventricular septal side. However, further tumor resection may have resulted in transmural resection of the ventricular septum; therefore, we opted for the limited resection range. Nonetheless, surgery was considered effective in preventing sudden death caused by tumor embolism or incarceration.

## Conclusions

Right ventricular metastasis of Ewing sarcoma is rare and has a poor prognosis; however, its resection may help prevent sudden death.

## Funding Support and Author Disclosures

The authors have reported that they have no relationships relevant to the contents of this paper to disclose.

## Appendix

Video 1 The video sequence begins with preoperative cardiac magnetic resonance and intraoperative transesophageal echocardiographic imaging. It then transitions to footage of the surgical resection of the right ventricular metastasis of Ewing sarcoma. The video concludes with postoperative transthoracic echocardiographic findings.
